# Inhibition of Fam114A1 protects melanocytes from apoptosis through higher RACK1 expression

**DOI:** 10.18632/aging.203712

**Published:** 2021-11-27

**Authors:** Miaoni Zhou, Fuquan Lin, Xingang Wu, Zhuyi Ping, Wen Xu, Rong Jin, Aie Xu

**Affiliations:** 1 The Third People’s Hospital of Hangzhou, Zhejiang, China

**Keywords:** melanocytes, fam114a1, RACK1

## Abstract

Fam114A1 is a gene closely related to the development of nerve cells, melanocytes, and nerve cells that originate from the neural crest of the embryonic ectoderm. Recent studies showed that Fam114A1 has a role in the occurrence of ankylosing myelitis spondylitis and autoimmune enteritis; still, its cellular function remains poorly understood. In this study, we investigated the effect of Fam114A1 on the biological activity of melanocytes. We found that the expression of Fam114A1 in vitiligo melanocytes (MCV-L, MCV-N, PI3V) was higher than that in normal melanocytes, and the biological function of melanocytes was significantly affected when the Fam114A1 gene was silenced. Inhibition of Fam114A1 increased proliferation, migration, and melanin synthesis proteins, decreased apoptosis, while its overexpression reversed this process. Mechanistically, we discovered that RACK1 is a target protein of Fam114A1 and that RACK1 can be negatively regulated by Fam114A1. Further study showed that Fam114A1 inhibition could not protect melanocytes from apoptosis once the expression of RACK1 protein was silenced. In summary, Fam114A1 is an effective regulatory protein for regulating the function of melanocytes. Inhibition Fam114A1 protects melanocytes from apoptosis through increasing RACK1.

## INTRODUCTION

The Fam114A1 gene is located on human chromosome 4, and it contains 14 exons [1–4]. Its encoded protein, also known as nervous system overexpressed protein 20 (NOXP20), is mainly expressed in the cytoplasm. Studies have shown that Fam114A1 protein is highly expressed in the mouse brain, spinal cord, and other nervous systems [1]. It is also differentially expressed during different stages of embryonic development, suggesting that Fam114A1 is closely related to the development of nerve cells [1]. Over recent years, genetic analysis has shown that Fam114A1 has a role in the occurrence of ankylosing myelitis spondylitis and autoimmune enteritis [5]; yet, its cellular function is not fully understood.

Melanocytes and nerve cells originate from the neural crest of the embryonic ectoderm [6–8]. The neural crest stem cells in the bulge region of the hair follicle can differentiate into neurons, glial cells, and melanocytes [9, 10]. Previous studies have implied that neurogenic factors may induce skin diseases or accelerate disease progression [11–13]. Dysregulation in differentiation, development, and melanocytes' activity can cause a series of skin pigmented diseases, such as generalized dyschromatopsia and mottle disease [14, 15]. Whether Fam114A1 is involved in the development and functional regulation of melanocytes has yet to be reported.

Receptor for Activated C Kinase 1 (RACK1) is a highly conserved intracellular adaptor protein with a molecular weight of about 35KDa, also known as the Guanine Nucleotide- binding protein subunit beta-2-like 1 (GNB2L1) [16]. It is a core ribosomal protein of the eukaryotic small (40S) ribosomal subunit [16–19]. Originally identified as an intracellular protein receptor for protein kinase C (PKC) [16], RACK1 is involved in regulating eukaryotic translation (IRES-mediated translation of viruses [20]) and ribosome quality control activities (nonfunctional 18S ribosomal RNA decay [21] and frameshifting at CGA codon repeats [22]). Recent studies have shown that RACK1 is a key scaffold protein that can interact with a variety of signaling proteins, such as protein kinases, phosphatases, ion channels and membrane receptors [23–25]. In addition, scaffold proteins may also interact with G proteins, IP3 receptors, apoptosis-related molecules, and structural proteins [23].

Fam114A1 contains the caspase recruitment domain (CARD) and may be involved in cell apoptosis [1]. In this study, we investigated the effect of Fam114A1 on the biological activity of melanocytes. We discovered that Fam114A1 is closely related to the growth of vitiligo melanocytes by affecting the proliferation, apoptosis, migration, and melanin synthesis of melanocytes and has a reverse effect on the expression of RACK1 protein. We also confirmed that RACK1 has an important role in regulating Fam11A1-induced apoptosis.

## MATERIALS AND METHODS

### Cell culture

In order to confirm that Fam114A1 is related to the activity of melanocytes, we compared the expression of Fam114A1 in the melanocytes of normal individuals (MC) and patients with vitiligo (MCV). Vitiligo is a typical skin disease characterized by impaired melanocyte function [12, 26–28].

Skin specimens for melanocyte culture were provided by the Third People’s Hospital of Hangzhou. Methods for isolating and cultivating cells from the specimens were previously described [29–32]. Specimens were washed twice with Hanks' solution (SIGMA, USA), then digested with 0.25% trypsin solution and 0.02% EDTA(SIGMA, USA) at 37° C for 30min, and cells were separated under an anatomical microscope. Suspension cells in F12 medium (GIBCO) containing 20 ng/mL bFGF(Pepro Tech), 20 ng/mL IBMX(SIGMA), 10 ng/mLCT(SIGMA), 50 ng/mL Gentamicin (GIBCO) and 10% fetal bovine serum (GIBCO) were placed in culture flask. The cells were cultured in a 5% CO2 incubator. After 3 days, geneticin (SIGMA) was added in the culture medium to eliminate the contamination of fibroblast and keratinocyte. The research protocol was approved by the Ethics Committee of Hangzhou Third People's Hospital.

### Inhibition and overexpression of Fam114A1 protein

The following plasmids were used to overexpress or knockdown the Fam114A1 protein: Fam114A1 overexpression plasmid (sense: 5'-CTAGCTAGCATGTCTGATGATGCTGGTG AC-3'; anti-sense: 5'- TGTCGCACCGGTCGGCTGTGCTTTCAAACAACT-3') and suppression plasmid (top:5’-GATCCGCAGAAGTGCATTGTGATTCAGGTACCTGAAT CACAATGCACTTCTGCTTTTTC- 3’; bot: 5’- TCGAGAAAAAGCAGAAGTGCATTGTGATTCAGGTACCTGAATCACAATGCACTTCTGCG3’).

The lentivirus with plasmid was coated and transfected into MCs. MCs transfected with suppression lentivirus (sh-Fam), and overexpression lentivirus (Fam) were separated into inhibition group and overexpression group, and MCs transfected with empty lentivirus was used as the control group (GFP). One day before the lentivirus infection, 2 × 105 MC cells were incubated in a 24-well plate. When the cell fusion reached 60% ~80%, we added the virus solution (multiplicity of infection MOI = 20) with 8 μg/mL DMEM medium with hexamethylene bromide and ammonium for 24 h. Cells were then cultured in a complete DMEM medium without hexamethonium bromide for an additional 48h, after which the transfection efficiency was analyzed with a fluorescence microscope. The expression of Fam114A1 was examined using RT-PCR and Western blot.

### MTT assay

Fam114A1 knockdown/overexpression cells and the control cells (2000 cells per well) were seeded in 96-well plates with 5 wells per group. After 48h, 10μl (10 mg/mL) of sterile MTT dye was added to each well and incubated at 37° C for 4 h. After removing the medium, 200 μl DMSO solution was added to each well and mix well for 10 min, then determine the absorbance at 570 nm with a microplate reader.

### Mitochondrial membrane potential detection

Cells were digested with 0.25% trypsin. Then, 5×10^4^-10^5^ resuspended cells were centrifuged at 1000 rpm for 5 minutes, after which the supernatant was discarded. Cells were then mixed with 100 μL of JC-1 working solution/per well, after which the cells were incubated for 15-20 mins in an incubator at 37° C. Finally, we resuspended cells with the fluorescent protective agent and performed detection with a flow cytometer.

### Immunohistochemistry

The skin tissue was fixed with 4% paraformaldehyde at room temperature for 20 minutes and embedded in paraffin. The tissue slides were preheated in an oven at 60° C for 2 h. Soak in xylene (I) at room temperature for 30 minutes, followed by 10 minutes in xylene (II). Slices were then soaked in 100%, 95%, 90%, 80%,70% ethanol and double distilled water for 10min, 5min, 2min, 2min, 2min, and 2min. After soaking in PBS-T, quenched with 3% peroxide-methanol at room temperature for 10 minutes. Slices were blocked with goat serum (1:100) at room temperature for 20 min, followed by rabbit anti-human Fam114A1 antibody (1:100) at 37° C for 2 h. After washing with PBS-T for 3 times, biotin-labeled secondary antibody (1:500, Abcam) was added and placed at 37° C for 30 min. Washed with PBS-T for 3 times again, and 3, 3-diaminobenzidine tetrahydrochloric acid was added, and incubated for 10 minutes at room temperature in the dark. The slices were stained with hematoxylin for 1 min at room temperature. The images were observed under a microscope.

### Immunofluorescence

The cells were fixed in 4% formaldehyde for 10min and incubated in 0.5% Triton X-100 for permeabilization. Cells were then incubated in 10% normal goat serum for 1h to block non-specific protein-protein interactions, and then with anti-Fam114A1, Rack1 antibody at 5μg/ml overnight at 4° C, and then with a secondary antibody (green, goat anti-mouse IgG (Abcam,) 1/250 dilution for 1h. Finally, DAPI was used to stain the cell nuclei (blue).

### Transwell assay

First, 10 μl of 0.5 mg/ml fibronectin was spread on the bottom of the transwell chamber. Then 10^5^ cells in a 1.5 ml EP tube were centrifuged at 200 g for 5 min to remove the supernatant. Cells were then mixed with 200 μl of fetal-free bovine serum DMEM medium and added to the chamber. DMEM medium containing 20% fetal bovine serum was added to the lower chamber. After 24 hours, the cells outside the chamber were fixed with methanol/glacial acetic acid (3:1) for 30 min and stained with crystal violet for 15 min. The membrane was washed and fixed on a glass slide and analyzed under the microscope (select 3 random fields (×100)). The average cell number in the three fields was used to calculate the number of migrated cells.

### Co-immunoprecipitation

Cells in the logarithmic growth phase were inoculated in T75 flasks (about 1.5 x 107 cells). After 24h, 3 mL (60 μl protease inhibitor cocktail) of Co-IP lysate/T75 flask was added after the cells were washed by PBS. The cells were then shaken at 4° C for 30 min and centrifuged at 12000 rpm for 10 min. A 200 μl of protein A and G agarose beads were then mixed and divided into 50 μl per tube. Then, after washing 3 times with the appropriate amount of Co-IP lysate, cells were centrifuged at 4,000rpm for 4 min. Then, 800 μl of Co-IP lysate was added to the beads overnight at 4° C after which, Fam114A1/RACK1 antibody and IgG (6 μg each) were added. The next day, a small amount of lysate was taken as input, and the pretreated cell lysate was first mixed with the antibody-coated beads and then placed at 4° C for 4 h. Next, we repeated the following for 4 times: centrifuge at 4000 rpm for 5 min, removing the supernatant, adding 1 mL of Co-IP lysate, turning for 5 min. After the last centrifugation, we let the cells stand for a few minutes, after which we discarded the supernatant. The samples were stored at -20° C until further use.

### Western blot

Epidermis and keratinocytes were lysed in denaturing RIPA lysis buffer supplemented with 20 mM N-ethylmaleimide (Sigma, USA), protease, and phosphatase inhibitors (Roche, Mannheim, Germany). SDS-PAGE (10%) was carried out using the mini-gel system from Bio-Rad. Proteins were transferred to a nitrocellulose membrane. The membrane was blocked with 5% non-fat milk in PBST buffer for 1 h at room temperature, then the membrane was incubated overnight at 4° C with specific primary antibodies against Fam114A1, RACK1, Bax, Bcl2, Caspase 3, cytc, P53, and GADPH/Actin (all 1:1000; Abcam, Cambridge, UK). The membrane was then washed with pBST buffer and incubated with fluorescent dye-labeled secondary antibody (Invitrogen, Carlsbad, CA, USA) for 1 h at room temperature in the dark. The proteins were visualized using an Odyssey Infrared Imaging System (LI-COR, Lincoln, Nebraska, USA).

### RT-PCR

Real-time fluorescent quantitative PCR was used to detect the expression of melanin synthesis-related genes TYR, TYRP-1, PMEL, MITF, DCT mRNA. The total RNA was extracted using Trizol. Then, 2 μg of total RNA was reverse transcribed into cDNA, and then subjected to fluorescence quantitative PCR detection. The primers were designed and synthesized by Shenggong Bioengineering (Shanghai) Co., Ltd., and the primer sequences were: GAPDH (F:5’-TGAAGGTCGGAGTCAACGGA-3’, R: 5’-CCTGGAAGATGGTGATGGGAT-3’), TYR (F:5’-GCAAAGCATACCATCAGCTCA-3’, R:5’-GCAGTGCATCCATTGACACAT-3’), TYRP1 (F:5’-TCTCTGGGCTGTATCTTCTTCC-3’, R: 5’-GTCTGGGCAACACATACCACT -3’), PMEL (F:5’-AGGTGCCTTTCTCCGTGAG-3’, R:5’-AGCTTCAGCCAGATAGCCACT-3’), MITF (F:5’-CTCACAGCGTGTATTTTTCCCA-3’, R:5’-ACTTTCGGATATAGTCCACGGAT-3’), DCT (F:5’-AACTGCGAGCGGAAGAAACC-3’, R:5’-CGTAGTCGGGGTGTACTCTCT-3’). The reaction system was 20 μl, and the reaction conditions: 95° C pre-denaturation for 2 min, 94° C denaturation for 1 min, 60° C annealing for 1 min, 72° C extension for 2 min, a total of 30 cycles. GAPDH was used as the internal control. The results are expressed as 2-ΔΔCt, ΔCt = Ct target gene-Ct internal reference gene, ΔΔCt = ΔCt overexpression group, or expression inhibition group-ΔCt control group; each sample was analyzed 3 times (the average was calculated).

### RACK1 silencing

RACK1 knockdown was done with siRNA (CAGGGATGAGACCAACTATGG) and Fugene 6 Transfection Reagent (Promega, E2691) according to the manufacturer’s instructions.

### Statistical analyses

We performed a one-way ANOVA to determine the statistical significance and a Dunnett’s post hoc test to independently compare the treatment groups with the control groups (SPSS). P<0.05 was considered statistically significant. All the data were presented as the mean ±SD unless stated otherwise.

## RESULTS

### Abnormal expression of Fam114A1 in melanocytes from vitiligo patients

**Figure 1:**
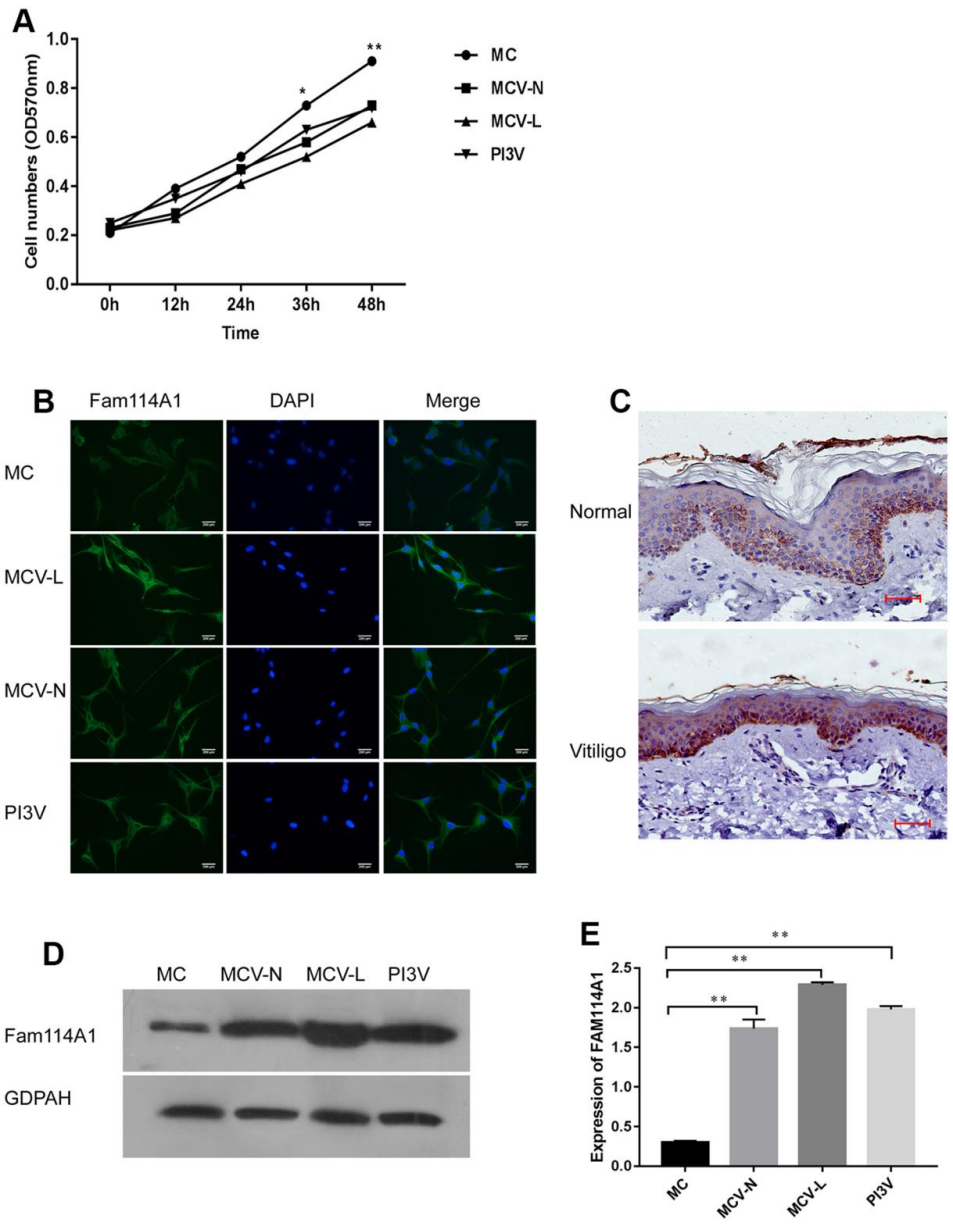
**Expression of Fam114A1 in the normal and vitiligo melanocytes.** (**A**) Comparing the growth rate of melanocytes from three different sources, it can be seen that the growth rate of melanocytes from around the skin lesions or normal parts of patients with vitiligo and the immortalized cell line PI3V of vitiligo are significantly slower than normal melanocytes. Statistical analyses were performed using one-way ANOVA and Dunnett’s post hoc test:*P<0.05, **P < 0.01 MC vs MCV-Lor MCV-N or PI3V. (**B**) The expression of Fam114A1 in melanocytes from different sources was detected by immunofluorescence (200×). (**C**) Immunohistochemical analysis of vitiligo skin lesions and normal human skins showed that Fam114A1 protein was highly expressed in the local skin lesions of vitiligo; (**D**, **E**) The expression of Fam114A1 in vitiligo melanocytes (MCV-L, MCV-N, PI3V) was confirmed by western blot, and significantly statistical results was shown. Statistical analyses were performed vs. MC using one-way ANOVA and Dunnett’s post hoc test: **P < 0.01 vs. MC.

In order to analyze the expression of Fam114A1 in melanocytes from different sources, we cultured melanocytes (see our previous studies [33, 34]) collected from the normal region of vitiligo (MCV-N), marginal lesion of vitiligo (MCV-L), the vitiligo cell line PI3V, and normal human melanocytes (MC). We found that the growth rate of cultured vitiligo-derived melanocytes MCV-N, MCV-L, and PI3V was significantly lower than that of normal melanocytes MC (all P<0.05, Figure 1A). Next, we used immunofluorescence technology to confirm the expression of Fam114A1 in melanocytes, and immunohistochemistry was performed to detect the expression of Fam114A1 in vitiligo epidermal tissues and normal skin tissues. The expression of Fam114A1 protein in vitiligo epidermal tissues was higher than that in normal skin tissue (Figure 1B, 1C). We also compared the expression of Fam114A1 protein in the normal region of vitiligo (MCV-N), the marginal lesion of vitiligo (MCV-L), and the vitiligo cell line PI3V and the melanocytes (MC) in normal people. The results showed that the expression of Fam114A1in MCV-N, MCV-L, and PI3V was significantly higher than that in MC; the highest expression was seen in MCV-L (all P<0.05, Figure 1D, 1E). The above results suggested that Fam114A1 was closely related to the growth of vitiligo melanocytes.

### Inhibition of Fam114A1 affects the function of melanocytes

**Figure 2:**
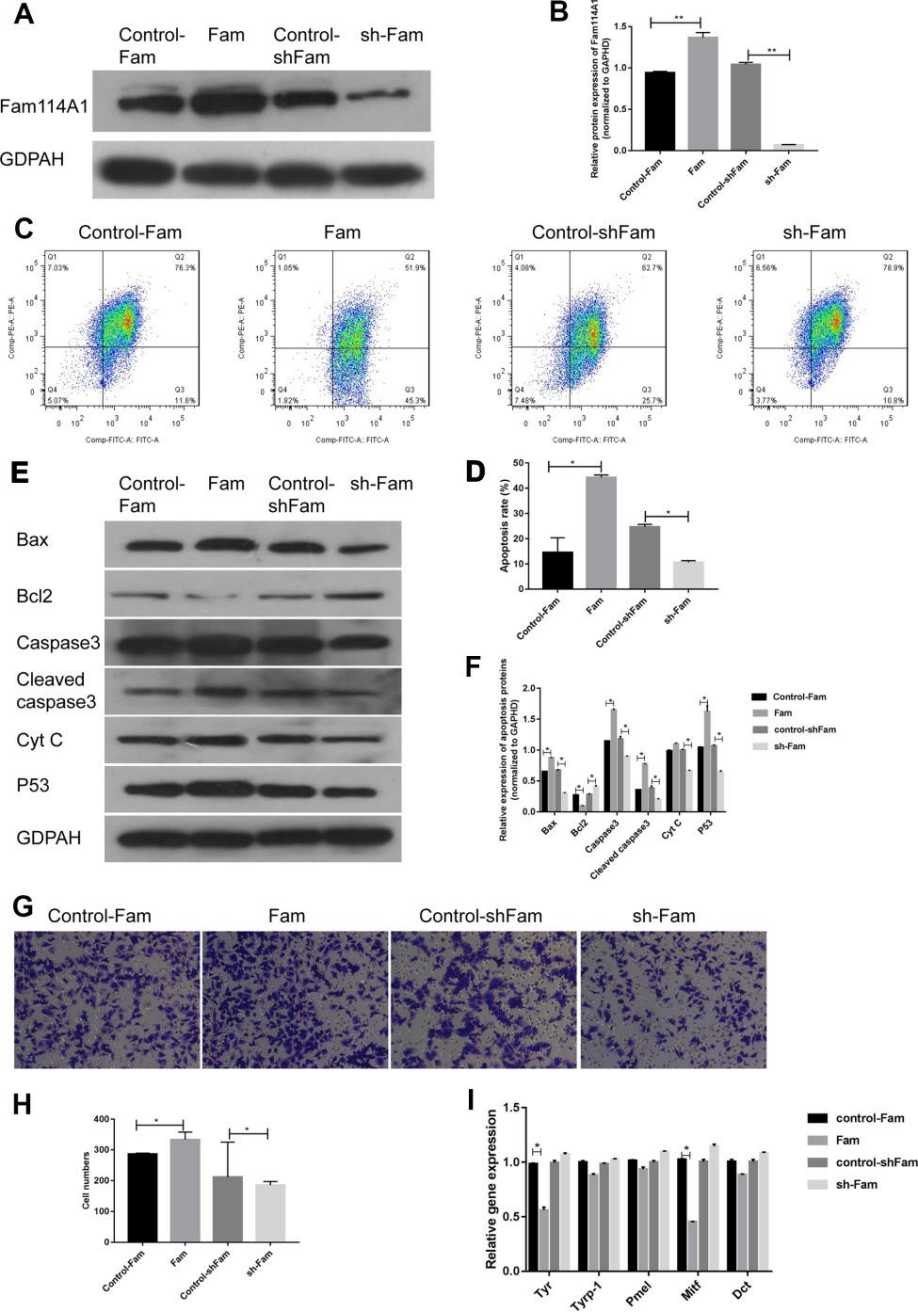
**Effects of Fam114A1 on the function of melanocytes.** (**A**, **B**) Western blot were used to detect the expression of Fam114A1 protein in MC after overexpression and suppression of plasmid transfection. The results showed that the expression of Fam114A1 protein in MC was successfully suppressed and overexpressed, Statistical analyses were performed using one-way ANOVA and Dunnett’s post hoc test: **P < 0.01 vs Control. (**C**, **D**) The effect of Fam114A1 on MC apoptosis was analyzed by mitochondrial membrane spot detection. It can be seen that when Fam114A1 protein is overexpressed, the apoptotic rate of MC increases significantly, and when the expression is inhibited, the apoptosis rate is dropped significantly. Statistical analyses were performed using one-way ANOVA and Dunnett’s post hoc test: *P < 0.05 vs Control. (**E**, **F**) Western blot was used to detect the expression of apoptosis-related proteins. It can be seen that the expression of apoptotic proteins bax, caspase3, cleaved-caspase3, and P53 decreased significantly in MCs inhibited by Fam114A1 protein, while the expression of apoptosis inhibitor bcl2 was significantly increased. Statistical analyses were performed using one-way ANOVA and Dunnett’s post hoc test: *P < 0.05 vs Control. (**G**, **H**) The migration of melanocyte was confirmed with transwell. activity. The migration of melanocyte was inhibited in sh-Fam MC compared with its control, Statistical analyses were performed using one-way ANOVA and Dunnett’s post hoc test: *P < 0.05 vs Control. (**I**) The expression of melanin synthesis proteins was detected by RT-PCR, the decrease of TYR and MITF was observed in sh-Fam MC vs its control, Statistical analyses were performed using one-way ANOVA and Dunnett’s post hoc test: *P < 0.05 vs Control.

Next, we examined the effect of Fam114A1 in Fam114A1-overexpressed MC (Fam), Fam114A1-downregulated MC (sh-Fam), and their respective control (Control-Fam and Control-shFam) by western blot. Cell apoptosis was detected by the measurements of mitochondrial membrane potential. The results showed that Fam114A1 was successfully over-expressed and down-regulated in Fam and sh-Fam groups, respectively (Figure 2A, 2B). In the Fam group, the apoptosis rate of melanocytes was higher than that of the control group (P<0.05, Figure 2C, 2E). Further detections of apoptosis-related proteins revealed that the expressions of Bax, caspase 3, cleaved-caspase3, Cytochrome C, and p53 were significantly increased, while the expression of Bcl2 was decreased, and the ratio of Bax/Bcl2 was significantly higher in Fam group melanocyte compared with the control group (all P<0.05, Figure 2D, 2F). Conversely, the above results were reversed in the sh-Fam group melanocyte. These results suggested that high expression of Fam114A1 could increase melanocyte apoptosis, while Fam114A1 silencing could protect melanocytes from apoptosis.

Transwell migration experiments confirmed that the migration ability of melanocytes decreased after Fam114A1 protein expression was inhibited (Figure 2G, 2H), and there were decreases in the expression of melanin synthesis related proteins MITF (microphthalmia-associated transcription factor) [35, 36] and tyrosinase (TYR) [37, 38] in melanocyte of Fam group (Figure 2I). Overall, these data suggested that Fam114A1 protein can affect the apoptosis, migration, and melanin synthesis of melanocytes.

**Figure 3:**
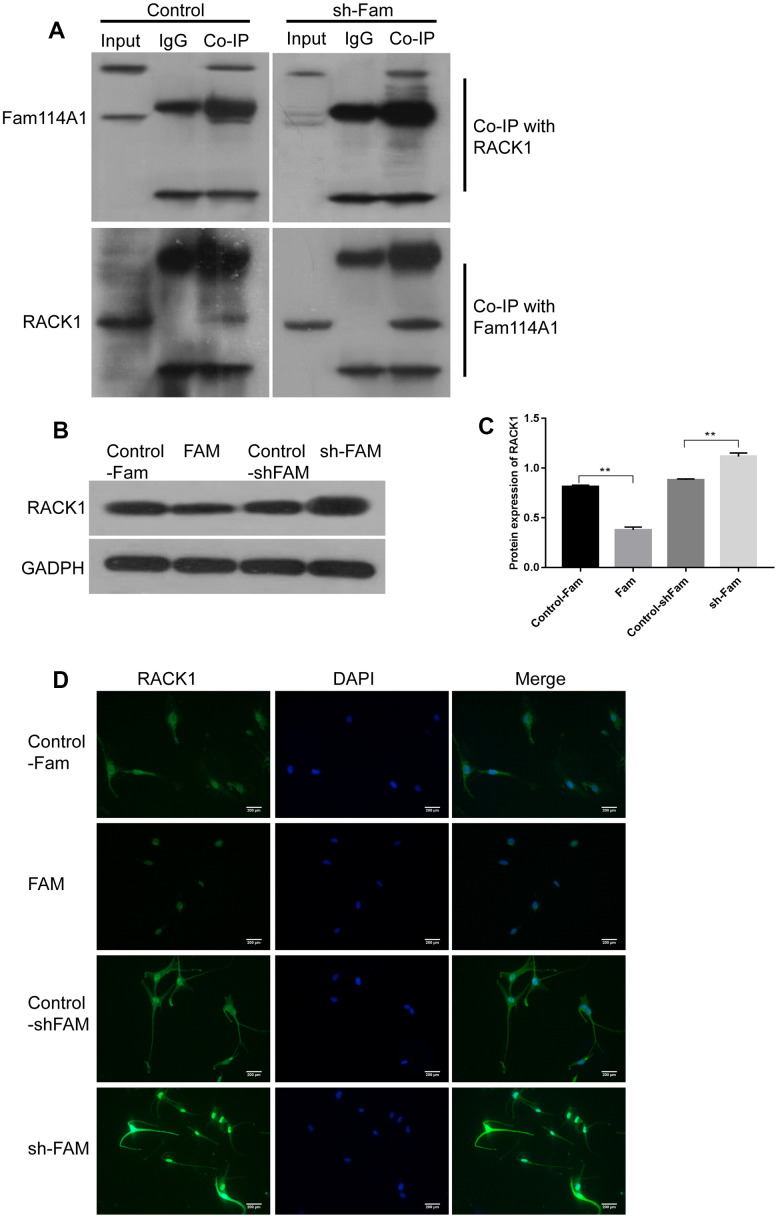
**RACK1 is the regulatory target protein of Fam114A1.** (**A**) In the control and sh-Fam groups, regardless of whether RACK1 or FAM1141 antibody was used for Co-IP, the mutual binding of RACK1 and Fam114A1 was observed, and it was observed that after the expression of Fam114A1 protein was inhibited, the co-precipitated RACK1 protein increased instead. (**B**, **C**) The expression of RACK1 in melanocyte of Fam, sh-Fam and their controls was detected by western bolt, the expression of RACK1 protein is negatively regulated by Fam114A1 protein. Statistical analyses were performed using one-way ANOVA and Dunnett’s post hoc test: **P < 0.01 vs Control. (**D**) The immunofluorescence detection of RACK1, the negatively regulation between Fam114A1 and RACK1 was confirmed once again.

The receptor for activated C kinase 1 (RACK1) is one of the regulatory target proteins of Fam114A1 in human melanocytes. RACK1 is a multifaceted scaffolding protein, a key mediator of various signaling pathways [39]. It adopts a seven-bladed β-propeller structure to facilitate the binding of various scaffolding proteins [39]. In this study, we found that Fam114A1 protein and RACK1 protein can bind and possibly interact with each other (Figure 3A, 3B). Therefore, we analyzed the effect of Fam114A1 inhibition or overexpression on RACK1 protein. We found that the expression of RACK1 protein (which is normally at the low expression in the cytoplasm) becomes highly expressed upon inhibition of Fam114A1 and is localized in the nucleus (Figure 3C, 3D). We confirmed that Fam114A1 has a reverse effect on the expression of RACK1 protein (Figure 3C, 3D).

### The role of RACK1 in Fam114A1 regulating apoptosis of melanocytes

**Figure 4:**
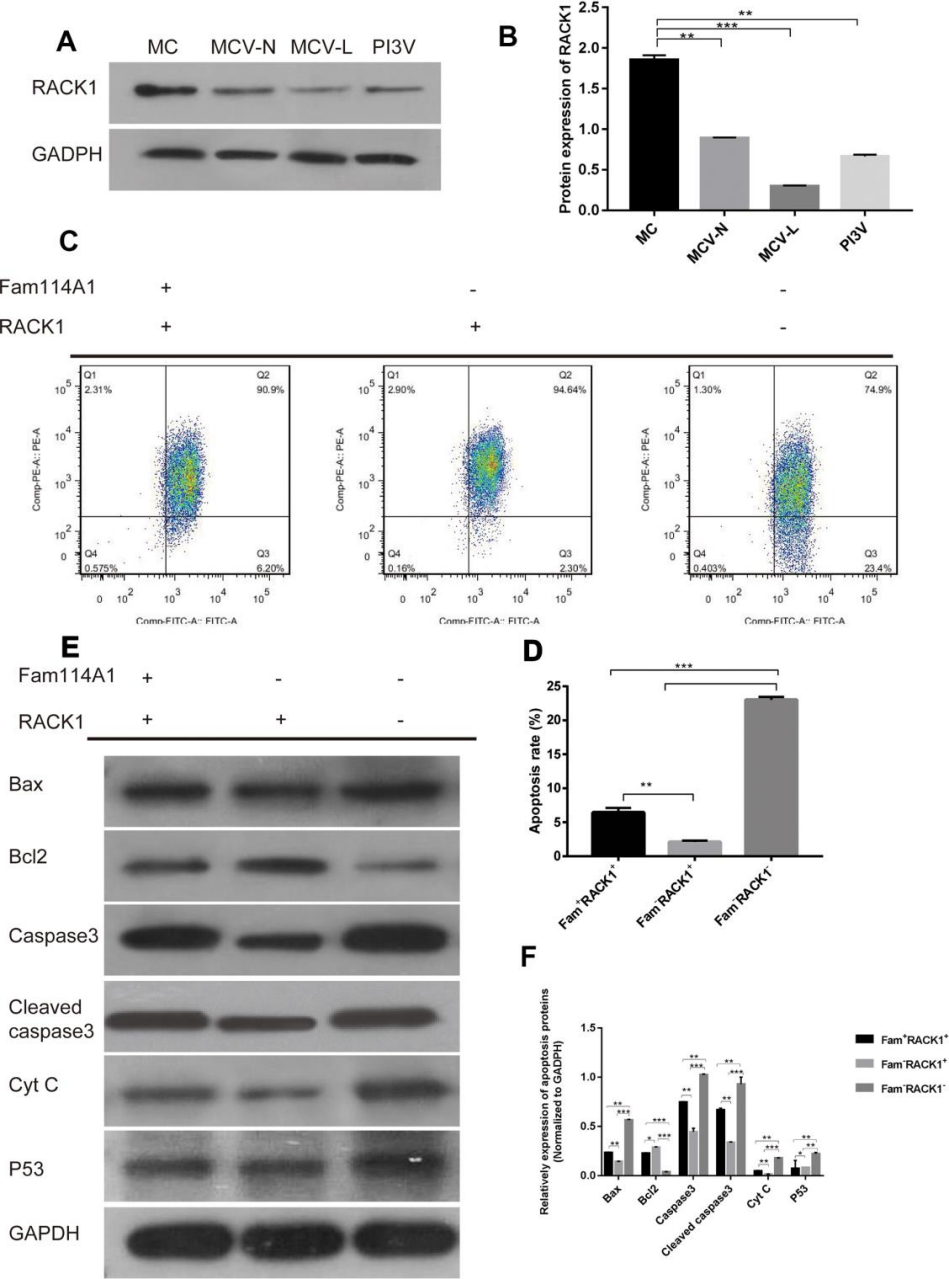
**The role of RACK1 in Fam114A1 regulating MC apoptosis.** (**A**, **B**) Expression of RACK1 protein in MC from different sources, the expression of RACK1 protein in vitiligo-derived MC is lower than that of normal MC. Statistical analyses were performed using one-way ANOVA and Dunnett’s post hoc test: **P < 0.01 vs MC; ***P<0.001 vs MC. (**C**, **D**) The apoptosis rate of Fam114A1-RACK1+ was decreased compared with Fam114A1+RACK1+, while the apoptosis rate of Fam114A1-RACK1- was increased compared with both Fam114A1+RACK1+ and Fam114A1-RACK1+. Statistical analyses were performed using one-way ANOVA and Dunnett’s post hoc test: **P < 0.01; ***P<0.001. (**E**, **F**) The expression of apoptosis-related proteins in Fam114A1+RACK1+, Fam114A1-RACK1+,RACK1and Fam114A1-RACK1- were detected by western blot, it can be seen that the expression of apoptotic proteins bax, caspase3, cleaved-caspase3, and P53 decreased significantly in Fam114A1-RACK1+ compared with Fam114A1+RACK1+ and increased significantly in Fam114A1-RACK1- compared with both Fam114A1+RACK1+ and Fam114A1-RACK1+, the result of bcl2 is just the opposite. Statistical analyses were performed using one-way ANOVA and Dunnett’s post hoc test: *P < 0.05, **P < 0.01, ***P<0.001.

Finally, we detected the expression of RACK1 protein in melanocytes from different sources (MC, MCV-N, MCV-L, PI3V) and found that in the melanocytes derived from vitiligo (whether it is around the skin lesion, the normal part of the patient, or immortalized cells) the expressions of RACK1 protein were significantly lower than that of normal melanocytes (P<0.05, Figure 4A). On the contrary, the expression of Fam114A1 protein was high.

We then constructed cells with inhibited RACK1 or Fam114A1 protein expressions. We found that reduced melanocyte apoptosis in cells when Fam114A1 was inhibited, as cells with inhibited both Fam114A1 and RACK1 expressions were not protected from apoptosis (Figure 4B, 4C). Thus, these results suggested that RACK1 has an important role in regulating Fam11A1-induced apoptosis.

## DISCUSSION

Fam114A1 is an uncharacterized gene with multiple splicing variants. Studies have predicted that six Fam114A1 variants can form stable mRNA transcripts and the most abundant one is derived from fifteen exons and encodes a protein with 563 amino acid residues (59 kDa) [5]; the second isoform contains a supplementary in-frame 1431 bp between exons 3 and 4. Fam114A1 encodes protein NOXP20, which contains a CARD23 and is mainly detected in the cytoplasm of transfected NIH3T3 cells [1]. NOXP20 protein contains CARD domain and is located in the proliferative regions of embryonic neurons as well as in the dividing regions of the adult brain, suggesting that NOXP20 protein plays a key role in regulating programmed cell death during central nervous system development and maintenance [1]. Current studies believe that there are many commonalities between neurological diseases and skin diseases. In some neurodegenerative diseases, such as sporadic Alzheimer's disease and Parkinson's disease (PD), increased ROS levels and decreased cell viability have also been detected in dermal fibroblasts of patients. In addition, several genes involved in central nervous system aging have also been found to be closely associated with skin aging in humans. Because patients with neurodegenerative amyloidosis (mainly AD, PD, and ALS) often have concurrent skin physiological changes, brain-skin linkages have been proposed as a relevant element in neurodegenerative studies [40]. It is well known that the neural crest stem cells in the bulge region of the hair follicle can differentiate into neurons and glial cells as well as melanocytes [5].

In addition, there are interlinked biological regulation of melanocytes and peripheral neurons, especially those associated with biological aging processes, and a reverse association between nevus number and the risk of memory loss has recently been reported. Melanocytes have been suggested as an *in vitro* cell model for AD because melanocytes share embryonic origin with nerve cells and are easier to biopsy than nerve cells [40]. Vitiligo is defined as a common de-pigmentary disease characterized by the destruction of melanocytes in the epidermis [26, 41–43]. Apoptosis of melanocytes is the main cause of vitiligo [26, 41–43]. Accumulated genetic and epigenetic changes may increase the chances of melanocytes being injured. Melanocytes can also be destroyed by excessive UV irradiation, hydrogen peroxide (H2O2), and inflammatory factors (all of which are defined as vitiligo risk factors) [28, 44, 45]. Also, it is possible that perturbations in apoptosis signaling pathways contribute to the loss of melanocytes [30, 41, 46, 47]. According to latest research findings, Vitiligo melanocytes share a common mitotic abnormality in neurodegenerative diseases. Interestingly, an important target for autoantibodies in vitiligo patients, Pmel17 (also known as GP100), belongs to the amyloid family. [40] Therefore, we speculate that Fam114A1, an important nervous system regulatory factor, may be involved in the loss and death of melanocytes in vitiligo.

In our study, we found that the growth speed of melanocytes from patients with vitiligo was significantly slower compared to melanocytes from healthy individuals. Fam114A1 is highly expressed in the skin of vitiligo skin lesions and is also highly expressed in melanocytes at the edges of the skin lesions and normal parts of the patient. To study the relationship between high expression of Fam114A1 and slow growth of melanocytes, we constructed melanocytes with inhibition and overexpression of Fam114A1 (sh-Fam and Fam, respectively). Our results showed that the overexpression of Fam114A1 protein could significantly increase the apoptotic rate of melanocytes and decrease the expression of melanin synthesis-related proteins TYR and MITF. While, the migration ability of melanocytes increased when Fam114A1 was over expressed. The high expression of the protein may be related to the apoptosis of local melanocytes. Fam114A1 is a functional factor that regulates the function of melanocytes.

In order to further study the possible mechanism of fam114A1 protein regulating the function of melanocytes, we used co-immunoprecipitation analysis to find proteins that interact with Fam114A1. RACK1 protein is one of the proteins that bind to Fam114A1 protein. RACK1 is a multifunctional scaffold protein involved in various pivotal cellular processes [39, 48–51]. It is clear that RACK1 protein is important in regulating cell activity, and its expression promotes cell growth and inhibits apoptotic ability [39, 48–51]. RACK1 is downregulated in aged human colonic epithelium and senescent NIH/3T3 cells, while its inhibition can accelerate cell senescence [52]. As the irreversible loss of proliferation characterizes senescence and apoptosis [53, 54], high RACK1 expression may be involved in the pathogenesis of many cancers [23]. This study confirmed that RACK1 is a binding protein of Fam114A1 and is negatively regulated by Fam114A1.

According to recent researches, vitiligo is frequently described as a disease associated with premature skin senescence. It has been reported that the age-related markers of melanocytes in the epidermis and dermis of patients with vitiligo are ubiquitously abnormal. Even melanocytes from normal skin exhibit an age-like phenotype *in vitro*, and this aging is associated with the abnormal activation of MAPK and p53 due to continuous intracellular ROS production [40]. In addition, hormonal levels are important in the development of vitiligo, while RACK1 expression is heavily dependent on hormonal levels. According to these theories, we believe that the negative regulation of RACK1 protein through Fam114A1 protein may have a role in the pathogenesis of vitiligo.

In this study, we detected the expression of RACK1 in melanocytes derived from the vitiligo skin lesions and normal parts and the vitiligo melanocyte cell line PI3V and found that the expression of RACK1 in melanocytes derived from vitiligo was significantly decreased compared with the normal melanocytes. This result was contrary to the high expression of Fam11A1 protein, and was consistent with the negative regulation mechanism of the two proteins.

According to the previous description in this article, the knockdown of Fam114A1 protein can protect melanocytes from apoptosis *in vitro*. We further knocked down the expression of RACK1 protein in melanocytes knocked down by Fam114A1 protein, and the results showed that the apoptosis of melanocytes was significantly increased. In combination with the knockdown of Fam114A1 protein in melanocytes, the expression of RACK1 protein was significantly up-regulated, suggesting that the knockdown of Fam11A1 and the negative regulation of RACK1 protein had an important role in the protection of melanocytes from apoptosis.

## CONCLUSIONS

In summary, the Fam114A1 protein can affect the apoptosis, migration and melanin synthesis ability of melanocytes, may be involved in the death of melanocytes in vitiligo, and play a certain role in the pathogenesis of vitiligo. RACK1 protein is the binding protein of Fam114A1, which can be negatively regulated by Fam114A1 protein, and plays a role in Fam114A1 regulating melanocyte apoptosis.
